# The Impact of Nanomaterials on Photosynthesis and Antioxidant Mechanisms in Gramineae Plants: Research Progress and Future Prospects

**DOI:** 10.3390/plants13070984

**Published:** 2024-03-29

**Authors:** Ping Li, Yunfei Xia, Kai Song, Duo Liu

**Affiliations:** School of Life Science, Changchun Normal University, Changchun 130032, China; chunlps@163.com (P.L.); xiayunfei1103@163.com (Y.X.)

**Keywords:** nanomaterials, Gramineae plants, photosynthesis, chlorophyll, oxidative stress

## Abstract

As global food security faces challenges, enhancing crop yield and stress resistance becomes imperative. This study comprehensively explores the impact of nanomaterials (NMs) on Gramineae plants, with a focus on the effects of various types of nanoparticles, such as iron-based, titanium-containing, zinc, and copper nanoparticles, on plant photosynthesis, chlorophyll content, and antioxidant enzyme activity. We found that the effects of nanoparticles largely depend on their chemical properties, particle size, concentration, and the species and developmental stage of the plant. Under appropriate conditions, specific NMs can promote the root development of Gramineae plants, enhance photosynthesis, and increase chlorophyll content. Notably, iron-based and titanium-containing nanoparticles show significant effects in promoting chlorophyll synthesis and plant growth. However, the impact of nanoparticles on oxidative stress is complex. Under certain conditions, nanoparticles can enhance plants’ antioxidant enzyme activity, improving their ability to withstand environmental stresses; excessive or inappropriate NMs may cause oxidative stress, affecting plant growth and development. Copper nanoparticles, in particular, exhibit this dual nature, being beneficial at low concentrations but potentially harmful at high concentrations. This study provides a theoretical basis for the future development of nanofertilizers aimed at precisely targeting Gramineae plants to enhance their antioxidant stress capacity and improve photosynthesis efficiency. We emphasize the importance of balancing the agricultural advantages of nanotechnology with environmental safety in practical applications. Future research should focus on a deeper understanding of the interaction mechanisms between more NMs and plants and explore strategies to reduce potential environmental impacts to ensure the health and sustainability of the ecosystem while enhancing the yield and quality of Gramineae crops.

## 1. Introduction

**Figure 1:**
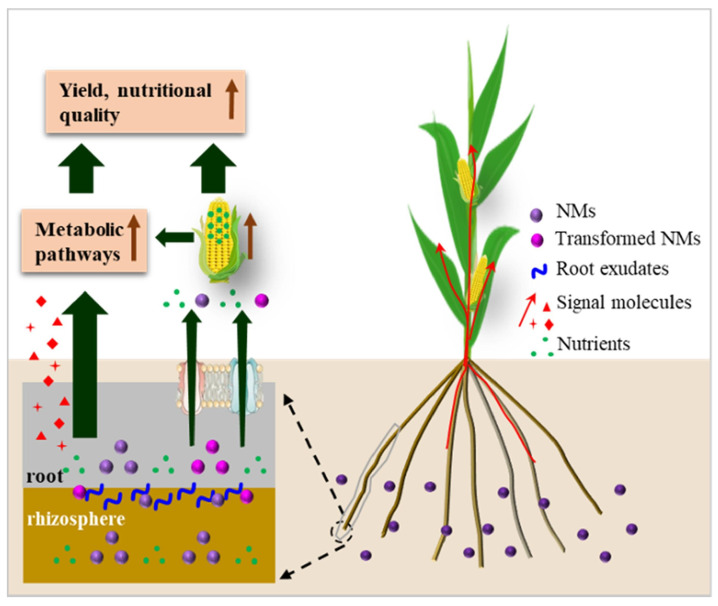
Signal transduction pathways in Gramineae plants influenced by NMs [3].

In recent years, the application of nanomaterials (NMs) in agriculture has been increasing, particularly in enhancing crop growth and stress resistance, improving grain nutritional value, and increasing yield. These materials, due to their small size, large surface area, and high reactivity, undergo a series of complex reactions in the soil, such as migration, transformation, dissolution, precipitation, dispersion, aggregation, and redox reactions [1]. These reactions not only affect the physicochemical properties of the soil but also have a significant impact on plant growth and development. The behavior of NMs in the soil primarily influences plants through their interaction with them. Studies show that NMs can adsorb on plant roots, promoting their entry into the cell wall and absorption by cells, thereby affecting plant growth and development [2]. The uptake process of NMs is commonly regarded as an active transport mechanism involving numerous additional cellular mechanisms, such as signaling, trafficking, and regulation of the plasma membrane. As shown in Figure 1, the biological response processes of NMs in enhancing the yield and nutritional quality of edible plants include (1) the plant’s response to NMs through signal transduction pathways and (2) NMs promoting the absorption and transport of nutrients [3]. While the impact of NMs on plants can be directly observed through growth indicators such as plant height and root length, physiological responses such as antioxidant enzyme activity and the photosynthesis system provide deeper insights, reflecting the complexity and depth of NMs’ interaction with plants [4].

Photosynthesis is the cornerstone of plant growth and development, with its efficiency directly influenced by chlorophyll content [5]. When the absorbed light energy exceeds the plant’s conversion capacity, harmful reactive oxygen species (ROS), such as superoxide anions and hydroxyl radicals, are produced [6]. ROS are predominantly generated in plant cells by chloroplasts, mitochondria, peroxisomes, and endoplasmic reticula, with chloroplasts being the principal source. These ROS significantly increase under environmental stresses, such as salinity, extreme temperatures, and water scarcity, affecting plant photosynthesis and overall health [7,8]. Therefore, there is a close relationship between a plant’s chlorophyll content and antioxidant enzyme activity, collectively reflecting the plant’s response to environmental stress and adaptive capacity. However, the mechanisms by which NMs affect plant photosynthesis and antioxidative responses remain unclear.

Current agricultural practices primarily rely on chemical fertilizers, which presents issues such as low nutrient efficiency in crops and negative environmental impacts. As an advanced nano-biotechnology, nanofertilizers exhibit superior transport characteristics in plant tissues/cells compared to traditional water-soluble fertilizers, demonstrating controllable migration rates [9]. Against this backdrop, studying the impact of NMs on Gramineae plants becomes particularly important. Gramineae plants, such as wheat, rice, and maize, are key to global food security. The unique properties of NMs, such as their small size and high reactivity, may have significant effects on the photosynthetic and antioxidant systems of these plants. Existing research suggests that certain NMs can enhance the activity of antioxidant enzymes within plants, thereby protecting chlorophyll and plant cells and mitigating ROS-induced oxidative stress [4]. This discovery reveals the potential role of NMs in enhancing plant stress resistance and growth efficiency. Furthermore, the impact of NMs is not limited to plants alone; their application also influences soil and soil microorganisms. Pawlet and others have suggested that the inhibitory effects of NMs are significantly influenced by soil properties [10]. Nevertheless, the excessive use of various NMs in agriculture and industry has led to their accumulation in soil, making it crucial to understand the relationship between their actual exposure concentrations and the biological effects on crops and symbiotic organisms [11].

Therefore, systematically studying and summarizing the impact of NMs on the photosynthesis and antioxidant systems of Gramineae plants holds significant scientific and practical value. This not only aids in understanding the mechanisms of NMs in plant physiological processes but may also offer new strategies for agricultural production to optimize growing conditions and improve yields, thereby contributing to global food security.

## 2. The Impact of Carbon-Based Nanomaterials on Gramineae Plants

**Figure 2:**
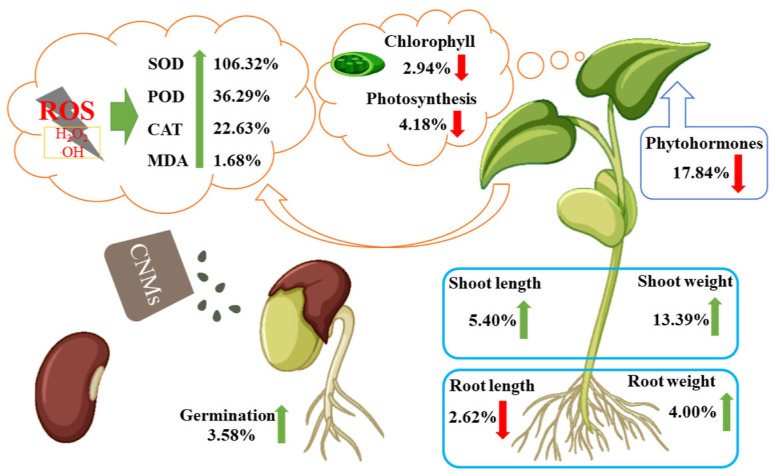
Impact of CNMs on plant photosynthesis and antioxidant enzyme systems [15].

Carbon, the most abundant element in the atmosphere, land, and oceans, is a fundamental constituent of life. The allotropy of carbon allows for a wide range of forms and structures in NMs, such as carbon nanotubes, graphene, and fullerenes (CNMs), all of which have been demonstrated to impact plant health [12]. CNMs can act as traditional fertilizers to promote seed germination and growth. However, activities like waste incineration and landfilling lead to the enrichment of CNMs in soil, inhibiting plant epigenetics and exhibiting cytotoxicity and genotoxicity [13,14,15]. Moreover, CNMs exhibit direct toxicity to plant-associated soil microorganisms, potentially altering the bioavailability of nutrients or modifying the toxicity of organic compounds and/or toxins. Whether this contributes to the impact of CNMs on plant photosynthesis requires further discussion. Current research indicates that the effects of CNMs on plants depend on the type, concentration, particle size, and environmental context of the NMs. Figure 2 shows that CNMs can enhance seed germination and seedling growth. Still, their presence inhibits root elongation and auxin activity, leading to reduced chlorophyll content and significantly increased antioxidant enzyme activity. The question of whether CNMs directly enter and are absorbed by plants or whether their interaction with soil and soil microorganisms affects plant growth and development has not been addressed or verified.

### 2.1. Graphene

Graphene and its derivatives, particularly oxidized graphene (GO), have garnered widespread attention in biological and environmental sciences. The complex, dual nature of GO’s impact on Gramineae plants has become a significant area of interest. GO has been found to promote seed germination and root growth, increasing antioxidant activity and chlorophyll content, possibly due to the enhanced permeability of seed coats, facilitating oxygen and water uptake and thus promoting seed metabolism and germination [16,17]. Observations under transmission electron microscopy and confocal fluorescence microscopy have shown that GO can spontaneously permeate membranes, leading to cell wall elongation and significantly aiding root elongation. Additionally, GO, with its abundance of polar hydrophilic groups, forms hydrogen bonds with water molecules, thereby enhancing soil water retention. Therefore, plants are generally treated through hydroponic methods to observe the transformation and impact of GO in plants. Utilizing the frictionless nature of GO surfaces facilitates water transfer to plant roots, promoting plant germination and growth [17]. However, this promotion is not without cost. For instance, He et al. showed that GO might cause oxidative damage in rice [16]. This effect may be attributed to GO partially covering the root surface, preventing the entry of mineral elements. GO internalization and deposition between the cell wall and plasma membrane inhibit the synthesis of chlorophylls a and b, damage chloroplast structure, and reduce photosynthetic efficiency in plants. Moreover, GO induces an imbalance in most nutrient elements in plants. The reduction in leaf contents of N, K, Ca, Mg, Fe, Zn, and Cu may be due to GO altering the root surface chemistry, affecting root–environment interactions. Additionally, Gabriela et al. found that the presence of GO inhibits the growth and photosynthesis of wheat, possibly due to GO accumulation in the roots, hindering nutrient uptake [18]. On the other hand, the impact of GO is significantly concentration-dependent. Zhang et al.’s study showed that different concentrations of GO treatment reduced chlorophyll content and photosystem II activity in wheat, suggesting that high concentrations of GO might inhibit plant growth and photosynthesis [19]. Similarly, Zhao et al. found that with increasing GO concentrations, chlorophyll content in maize seedlings initially increased and then decreased. In contrast, peroxidase activity and malondialdehyde content increased, indicating oxidative stress induced by GO at certain levels [20]. Unlike metal nanoparticles (MNPs), GO is highly stable, and its mechanism of inhibiting the growth of Gramineae plants is complex. At low concentrations, GO can enhance the synthesis of chlorophyll and even increase the activity of antioxidative enzymes secreted by the roots. At high concentrations, GO covering the root surface impedes the entry of mineral elements, leading to inhibited stem height and root length, reduced diameter of root cortical cells, cellular contraction and deformation, and induced oxidative stress in plants. Research by Ali et al. highlighted that the biological functions of NMs depend on their physicochemical properties, application methods, and concentrations. This study further confirmed the impact of nanomaterial concentrations on plant growth and adaptability [21]. Additionally, Hu et al. confirmed the potential toxicity of GO, indicating a reduction in chlorophyll content, damaged chloroplast structure, and increased ROS production despite enhanced antioxidant enzyme activity [22]. The enhancement of root elongation by GO does not reflect a positive effect on root growth, as increased GO concentrations result in shorter and sparser root hairs. These findings indicate that GO induces oxidative stress in plant roots. However, the effects of GO are not entirely negative. Yin et al. found that GO could mitigate the inhibitory effects of the heavy metal Cd^2+^ on the growth of rice roots and shoots [23]. In summary, the impact of GO on Gramineae plants is multifaceted, including inhibition of root growth and photosynthesis reduction, as well as enhanced antioxidant enzyme activity, thereby alleviating oxidative damage to some extent. GO induced responses thus inhibit nutrient absorption. However, current understanding of the impact of GO on plant nutrient components is still in its nascent stages, and the verification of whether GO accumulates internally and integrates into the seeds of plants remains unverified. These findings emphasize the importance of in-depth research on GO’s physiological impact on plants to better understand and utilize these NMs in agriculture and environmental protection. 

### 2.2. Carbon Nanotubes 

Apart from graphene and its derivatives, other CNMs, such as carbon nanotubes (CNTs), have also significantly impacted plants. CNTs are mainly classified into single-walled carbon nanotubes (SWCNTs) and multi-walled carbon nanotubes (MWCNTs), each with differing roles in plant physiological processes. Due to the insolubility of CNTs in water, they are typically prepared as suspensions for use in hydroponic cultivation, and their translocation is often achieved by adding them to the growth medium. Early studies found that CNTs could penetrate chloroplast membranes and stimulate light absorption, thus affecting plant photosynthesis [24]. Conversely, at low concentrations, CNTs enhance plant growth and development by facilitating the efficient absorption of water and essential nutrients (Ca and Fe). In particular, low concentrations of SWCNTs have been found to internalize and localize within the lipid bilayers of chloroplasts, significantly improving the photosynthetic performance of plants while reducing the formation of ROS, suggesting their crucial role in improving plant photosynthetic efficiency [25]. The effects of multi-walled carbon nanotubes (MWCNTs) are more complex. They can penetrate cell walls, forming artificial stomata and acting as additional nutrient transport channels [26]. Additionally, MWCNTs cause changes in the composition, stiffness, and permeability of root lipid membranes, increasing the transduction of plant water channel proteins, thus enhancing plant water absorption, seed germination, and seedling development [27,28]. However, high concentrations of MWCNTs severely inhibit root elongation and leaf development. Transcriptome data reveal that MWCNTs suppress plant auxin signaling and photosynthesis, while enhancing ROS, toxin metabolism, and plant responses to pathogens to combat the oxidative stress induced by MWCNTs [29]. Anjali et al.’s study found that rice treated with MWCNTs exhibited denser stomata and longer roots, which are beneficial for water and mineral absorption, accelerating plant growth [30]. However, the impact of MWCNTs on plants is concentration-dependent. At low concentrations, MWCNTs improve seedling growth indices and water content, especially in roots; at high concentrations, their effectiveness diminishes, possibly due to increased toxicity from nanotube aggregation [31]. Tan et al. investigated the toxicity of MWCNTs on rice cells, finding that the application of MWCNTs led to chromatin condensation, cell membrane detachment, decreased cell viability, and cell death [32]. Additionally, another study by Tan showed that with increasing concentrations of MWCNTs, cell density decreased [33].

High concentrations of MWCNTs might excessively absorb iron in plants, hindering chlorophyll production and increasing SOD and POD activity [34]. High concentrations of MWCNTs accumulate extensively at the radicle tips, demonstrating strong adsorption affinity towards Zn and Cu, impeding the translocation of Zn from cotyledons to seedlings and ultimately inducing oxidative stress in plants. Additionally, the excessive absorption of iron by these nanomaterials hinders chlorophyll production. To overcome the low solubility issues of SWCNTs and MWCNTs in water, researchers have developed water-soluble carbon nanotubes (wsCNTs). WsCNTs promote the growth of plant roots, branches, and offshoots with non-toxic properties, potentially aiding water resource optimization in arid regions [35]. Irina and colleagues explored the accumulation of MWCNTs from green algae through the food chain to consumers, indicating an increased risk of CNT accumulation in the food chain.

When discussing the impact of carbon-based nanomaterials, such as CNTs and graphene, on plants, it is essential to consider their potential risks thoroughly. These nanomaterials have shown tremendous potential in various applications due to their unique physicochemical properties, including promoting plant growth and protecting plants from diseases in the agricultural sector. However, as the use of these nanomaterials increases, their potential negative impacts on the environment have also drawn scientific attention. Firstly, carbon-based nanomaterials may adversely affect soil health. These nanomaterials can alter the physicochemical properties of soil, impacting the structure and function of soil microbial communities. For instance, carbon nanotubes have been found to affect the growth and metabolism of soil bacteria, potentially leading to changes in nutrient cycling [36]. Additionally, the introduction of graphene has been observed to affect soil moisture retention capacity and soil aeration, thereby impacting the plant growth environment. Secondly, the potential impact of carbon-based nanomaterials on water quality cannot be ignored. These nanomaterials may enter water bodies through agricultural runoff, affecting the health of aquatic ecosystems. Studies have shown that carbon nanotubes and graphene can exhibit high stability and the potential for long-term presence in aquatic environments, thereby potentially exerting toxic effects on aquatic organisms [37]. Lastly, the accumulation of carbon-based nanomaterials in the food chain is an important consideration. These nanomaterials can be absorbed and accumulated by plants, ultimately affecting the health of humans and other organisms. Research has indicated that plants can absorb and accumulate carbon nanotubes through their roots, which may lead to the transfer and biomagnification of nanomaterials in the food chain [38].

### 2.3. Nano-Biochar 

Biochar (BC), a carbon-rich solid produced through thermochemical pyrolysis, is commonly used as a soil amendment. Nano-biochar (Nano-BC) consists of nanoparticles synthesized during the carbonization process [39]. Nano-BC effectively regulates the mobilization and adsorption of micro- and macronutrients, including potentially toxic metals and hazardous pollutants like pesticides [40]. By attaching or depositing on the root surface and forming shell-like structures, they utilize their high adsorption affinity for heavy metals to significantly inhibit the influx of heavy metals into root cells, thereby reducing the negative impact of heavy metals on plants. Additionally, nano-BC regulates the chemical functional groups on the surface of organic compounds, enhancing root nutrient absorption [41,42]. Nano-BC not only reduces plant uptake of heavy metals but also alleviates the harm caused by invasive plants. Under treatment with root exudates from invasive species, applying nano-BC increases chlorophyll concentration, reduces oxidative stress and lipid peroxidation, and decreases negative gene expression at the molecular level [43,44]. Our review of the existing literature shows that most research on CNMs focuses on direct assessments of their physiological and biochemical effects on plants. However, the interactions between growth media in the environment, the duration of CNM exposure, species differences among receptors, and factors related to CNMs, such as type, synthesis conditions, concentration, and particle size, are complex. Progress in understanding the potential toxicity mechanisms at the molecular-genetic level is minimal, making it challenging to determine the consistency of CNMs’ effects on plants.

**Figure 3:**
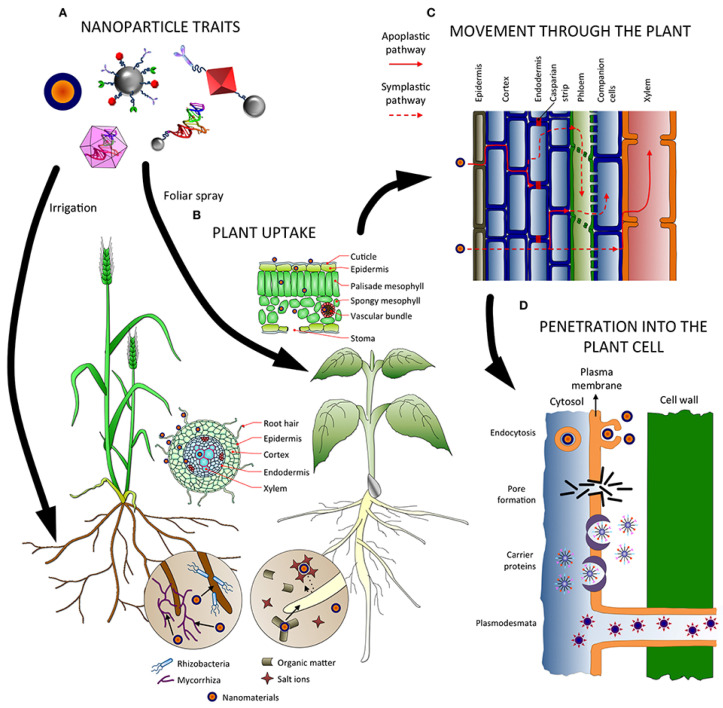
Factors influencing absorption, uptake, transport, and penetration of nanoparticles in plants. (**A**) Nanoparticle traits affect how they are taken up and translocated in the plant, as well as the application method. (**B**) In the soil, nanoparticles can interact with microorganisms and compounds, which might facilitate or hamper their absorption. Several tissues (epidermis, endodermis, etc.) and barriers (Casparian strip, cuticle, etc.) must be crossed before reaching the vascular tissues, depending on the entry point (roots or leaves). (**C**) NMs can follow the apoplastic and/or the symplastic pathways for moving up and down the plant and adradial movement for changing from one pathway to the other. (**D**) Several mechanisms have been proposed for the internalization of nanoparticles inside cells, such as endocytosis and pore formation, mediated by carrier proteins and plasmodesmata [45].

The transport and accumulation mechanisms of these carbon-based nanomaterials (CNMs) in cereal crops are crucial for understanding their impact on the plants’ photosynthetic systems. As illustrated in Figure 3, NMs make contact with plant root systems, entering the plant cell walls and root epidermal cells. Once NMs penetrate the plant, they can move through tissues via two pathways: the apoplast and symplast pathways. The apoplastic pathway is crucial for the radial movement within plant tissues, allowing NMs to reach the central cylinder and vascular tissues of roots and ultimately transfer to the plant leaves. Another significant symbiotic transport involves the phloem’s sieve elements, enabling distribution to non-photosynthetic tissues and organs. In the case of foliar application, NMs must bypass the barrier provided by the cuticle and enter along lipophilic or hydrophilic pathways. The movement of NMs within plants is crucial, as it indicates which parts of the plant they can reach and where they may end up and accumulate. For example, if nanoparticles are primarily transported through the xylem rather than the phloem, they will predominantly move from the roots to the stem and leaves rather than downward, suggesting that their application should be on the plant’s roots for good distribution within the plant. Conversely, if nanoparticles exhibit good mobility through the phloem, they should be applied via foliar spray. Moreover, NMs moving along the phloem may accumulate in plant tissues acting as sinks, such as fruits and grains [45].

In conclusion, CNMs typically penetrate root cells or adhere to roots, stimulating plant metabolism and biochemical reactions in plant cells. These materials significantly stimulate the photosynthesis process, affecting chlorophyll A, chlorophyll B, and carotenoid content, altering the rate of photosynthesis and plant metabolites through the Calvin cycle [46]. CNMs also stimulate water channel proteins, regulating root water absorption, acquiring more nutrients for photosynthetic synthesis, enhancing chlorophyll content and photosynthetic activity in vegetation, and significantly increasing antioxidant enzyme activity, thereby enhancing plant stress resistance and adapt ability. Plants treated with CNMs have higher elemental contents in their seeds. However, high concentrations of CNMs may interact with amino acids in biological cells, leading to CNM wrapping or adsorption around cells, slowing plant growth, and causing electrolyte leakage and protein and lipid oxidation. These materials may also disrupt the expression of genes related to chlorophyll synthesis, inhibiting plant photosynthesis.

## 3. The Impact of Metal Nanoparticles on Gramineae Plants

**Figure 4:**
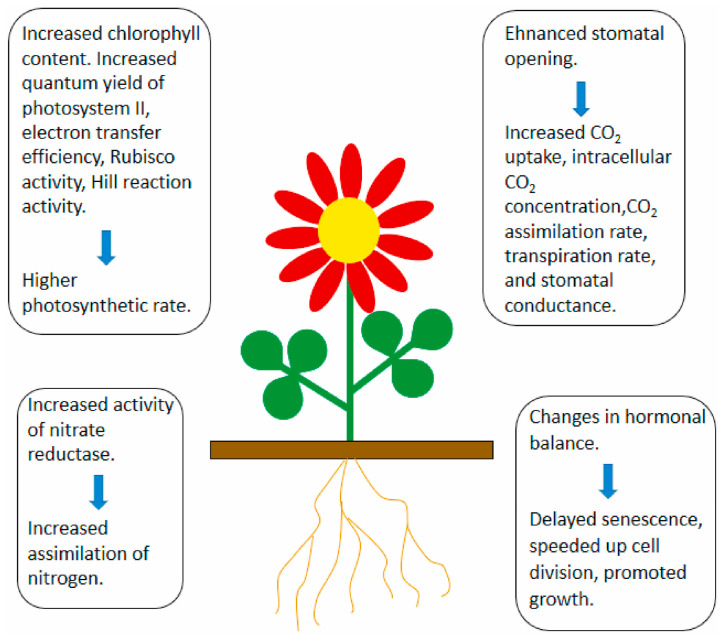
Effects of MNPs on plant growth and yield [48].

MNPs, with dimensions less than 100 nm in one axis, exhibit exceptional physicochemical properties, such as large surface areas, making them widely used in nanofertilizers, nanopesticides, etc. However, their easy absorption, transformation, and accumulation in plants and continuous release into the environment can lead to metal particle aggregation in certain tissues, impacting both plants and the environment. Earlier studies suggest that the harmful effects of MNPs on plants, primarily oxidative stress and damage, ultimately hinder growth [47]. More recent research indicates a positive role of metallic NMs in plant growth; for instance, Figure 4 shows MNPs enhancing stomatal density, chlorophyll, and enzyme activity, thus promoting plant growth [48]. NMs can enter leaf cells through the leaf epidermis or stomata, moving via the apoplastic or symplastic pathways.

### 3.1. Effect of Nanocerium on Gramineae Plants

Cerium, one of the rare earth elements, is not essential for plants, but cerium oxide nanoparticles (CeO_2_ NPs) positively affect photosynthesis and stomatal conductance at low concentrations [48]. Many studies on plant absorption of NMs have been conducted using aqueous suspensions or hydroponic media, with CeO_2_ NPs often being used in such research as they are considered to be stable and insoluble. Plants usually absorb and internalize CeO_2_ NPs by incorporating them into the soil. CeO_2_ NPs penetrate leaf surfaces through stomata, passing through mesophyll cell walls and plasma membranes to chloroplasts via a non-endocytic pathway influenced by mesophyll membrane potential [49]. CeO_2_ NPs can enter plant roots and even be transported to stems, indicating that they undergo biotransformation and are to some extent soluble. However, almost all available plant studies on CeO_2_ NPs have not considered the biotransformation and solubility of these NMs. CeO_2_ NPs have the capacity to absorb and release oxygen in surface-catalyzed redox reactions. At low concentrations, CeO_2_ NPs dissolve in their oxidized state, such that further Ce^4+^ can be reduced to Ce^3+^ and then be released, promoting the content of chlorophylls a and b and total chlorophyll, enhancing the plant’s capacity to scavenge ROS and boosting photosynthesis. The plasma effect can enhance the activity of the photosystem, thereby enhancing the photosynthesis of chlorophyll biomass. Simultaneously, CeO_2_ NPs form free radicals within the photosynthetic mechanism by altering the molecular structure of chlorophyll. However, high concentrations of CeO_2_ NPs reduce chlorophyll content and increase the activities of hydrogen peroxide and superoxide dismutase enzymes [50]. Under high concentrations of CeO_2_ NPs, the reduction in chlorophyll content may be due to excessive production of ROS and lipid peroxidation, potentially damaging chloroplast structures. The decline in chlorophyll is primarily caused by the degradation of chlorophyllase, disruption of chlorophyll structure, and instability of pigment complexes. Zhang et al. found that 500 mg/L or higher concentrations of CeO_2_ NPs induce lipid peroxidation and cell membrane damage in lettuce [51]. These studies indicate that high concentrations of CeO_2_ NPs may adversely affect plant dry weight, chlorophyll content, and nutrient availability, leading to oxidative stress. High concentrations of CeO_2_ NPs applied to the leaf surface might block stomata, reducing transpiration, photosynthesis, growth, and plant resistance [52]. However, other studies report no significant effect of CeO_2_ NPs on chlorophyll content in wheat leaves [53], while Wang et al. found that under hydroponic conditions, 100 mg/L CeO_2_ NPs significantly increases fresh root biomass in rice [54]. These discrepancies could result from varying plant species, concentrations, and durations of exposure to CeO_2_ NPs. 

To mitigate the adverse effects of CeO_2_ NPs, researchers have experimented with their combined use with other substances. For example, Yan et al. found that treatment with carbon dots (CDs) and CeO_2_ NPs under hydroponic conditions significantly increased the chlorophyll content and peroxidase activity in wheat [55]. This finding could be attributed to the combined application of CDs and CeO_2_ NPs increasing chlorophyll accumulation, enhancing plant utilization and absorption of sunlight, and improving the photosystem activity and photosynthesis in wheat. Azka et al. discovered that gibberellic acid (GA) could reverse changes caused by CeO_2_ NPs alone, enhancing plant chlorophyll content and antioxidant enzyme activity [56].

Overall, CeO_2_ NPs at low doses might positively impact the growth of Gramineae plants and the amino acid content in grains, acting as catalysts for chlorophyll formation and maintaining chloroplast structure, whereas high concentrations could block stomata, causing oxidative stress. Therefore, their combined use with other functional materials or plant hormones may be an effective way to reduce oxidative damage from CeO_2_ NPs.

### 3.2. Impact of Iron-Based Nanomaterials on Gramineae Plants

**Figure 5:**
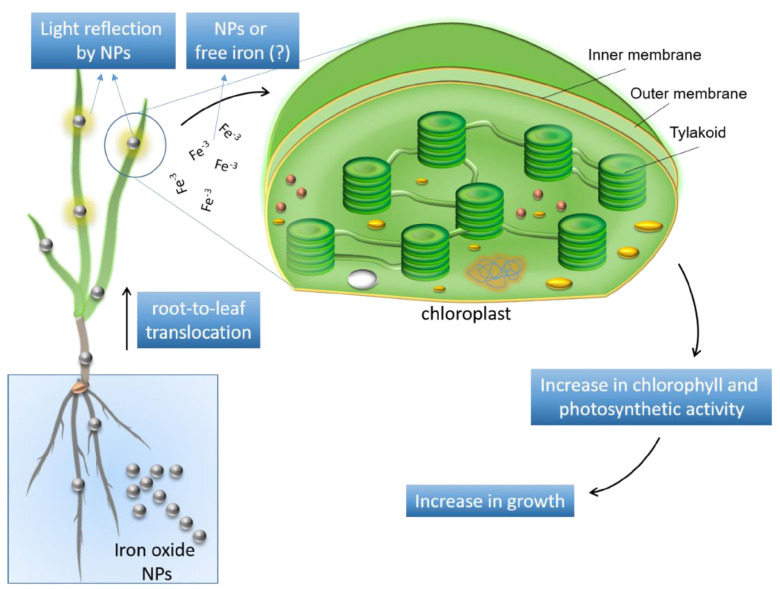
Absorption and response of plants to iron-based nanoparticles [59].

The positive effects of NMs on plants are believed to be related to their large specific surface areas, leading to high solubility and reactivity, which determine their effective interaction with membranes, other cell components, proteins, and lipids [57]. Iron is a vital micronutrient for plant growth, playing a critical role in chlorophyll synthesis, respiration, and redox reactions despite its low concentration in plants. Iron deficiency leads to chlorosis and affects photosynthetic efficiency (Figure 5). Usually, iron fertilizers are added to crops to supplement this element. There are typically two methods for iron to enter the root zone: various compounds or organic complexes and diffusion. When plants absorb iron, it migrates from higher to lower concentrations on the root surface. Chelated iron in the soil solution spreads to the roots through mass flow or diffusion. Iron is transported across the cell membrane, reduced, and released from the chelating molecule, with the inner epidermal cells and epidermal cells of roots absorbing iron. Before entering the xylem, iron is loaded into the endodermal cells’ pericycle sheath. The exodermis is where most of the iron is transported to the shoots. From this point, it can be transported across the cell’s plasma membrane into the cytoplasm and organelles [58].

Iron-based nanomaterials, much smaller than typical iron oxides or iron molecules, can form more complexes with different molecules, providing more bioavailable iron to plant organs. Similar to zinc and copper, iron-based nanomaterials are usually absorbed gradually, while their ionic forms are rapidly absorbed and immediately involved in various biochemical reactions [60]. Iron-based NMs, with their small surface areas and high biocompatibility, are considered effective iron fertilizers. These nanoparticles not only enhance crop antioxidant enzyme activity, promoting seed germination and seedling growth, but also increase chlorophyll content and prevent heavy metal accumulation. Kokina et al.’s study showed that barley seedlings treated with low concentrations of magnetite nanoparticles (Fe_3_O_4_ NPs) under hydroponic conditions exhibited significantly increased growth rates, chlorophyll contents, and specific miRNA expression levels. In contrast, high concentrations of Fe_3_O_4_ NPs showed a negative correlation with chlorophyll content [61]. Tombuloglu’s study revealed that under hydroponic conditions, Fe_3_O_4_ NPs penetrate and internalize in plant root cells, significantly boosting barley growth, chlorophyll content, and dry weight and doubling root length with increasing concentrations of Fe_3_O_4_ NPs [62]. Plants treated with Fe_3_O_4_ NPs also exhibit higher potassium and phosphorus contents in their leaves, these elements being crucial for maintaining the activity of many enzymes, including those involved in the Calvin cycle and dark respiration. Some SOD isozymes in plants depend on Fe activation, so an increased Fe content in leaves maximally reduces the adverse effects of ROS produced under high light conditions, thereby increasing chlorophyll content and enhancing the net assimilation rate of CO_2_. However, the effects of iron-based NMs are not uniform. Jie et al. found that high concentrations of nano-zero-valent iron (NZVI) inhibited the growth of rice seedlings, which showed clear symptoms of iron deficiency. However, toxicity symptoms decreased with aged nZVI [63]. Although nZVI can accumulate in the roots, it hardly transfers to the edible parts of the plant. The activation of H^+^-ATPase induced by nZVI, leading to proton secretion, may increase the availability of P in the soil by acidifying the rhizosphere. Overexpression of PM H^+^-ATPase enhances stomatal opening, promoting CO_2_ absorption and thus enhancing photosynthesis. However, overexpression of PM H^+^-ATPase-related genes (CsHA1) by nZVI affects plant growth and Fe uptake [64]. Conversely, Li et al. discovered that foliar application of iron-based nanoparticles, such as Fe and Fe_3_O_4_, significantly increased the chlorophyll content, net photosynthetic rate, and biomass in maize plants [65]. Saleha’s study further indicated that foliar application of appropriate concentrations of glutamic acid-modified trivalent iron nanoparticles (Glu-ZVFe NPs) and indole acetic acid (IAA) enhanced root length, leaf area, and germination rate in maize, reducing the toxic effects of lead ions [66]. Some Fe_3_O_4_ NPs found in chloroplast thylakoids may envelop chloroplast surfaces in the Fe_3_O_4_ NP–chloroplast system, acting as both electron donors and acceptors instead of releasing iron, enhancing photosynthesis.

The effect of hematite NMs, another type of iron-based NMs, is also noteworthy. Tombuloglu’s study using hematite nanoparticles (Fe_2_O_3_ NPs) on barley showed toxicity inhibiting germination and pigment synthesis [67]. Lu et al. found that foliar application of Fe_2_O_3_ NPs on wheat seedlings led to excessive production of OH- in plant bodies, accelerating chlorophyll degradation and significantly reducing photosynthesis [68]. Foliar-applied Fe_2_O_3_ NPs mostly accumulate in the leaves, with a portion being translocated to the stem and roots through the vascular system. It is noteworthy that Fe_3_O_4_ NPs might bind to organic acids or ligands secreted by leaf surfaces [57]. Additionally, Elena et al. reported positive effects of nanohydroxyapatite (nHA) and Fe_2_O_3_ NPs on photosynthesis in maize and winter wheat plants [69]. In another study, different sizes of Fe_2_O_3_ NPs absorbed by roots and transferred to leaves indicated that nanoparticles as an iron source in metabolic reactions could stimulate photosynthetic mechanisms, enhancing root length, plant height, biomass, and chlorophyll content in wheat [59]. Lastly, Iannone et al. found that hydroponically applied citrate-coated Fe_3_O_4_ NPs did not affect germination rate, chlorophyll content, or plant growth in wheat while enhancing antioxidant enzyme activity in the roots and aerial parts, showing a response against oxidative damage [70]. Upon absorption by plant roots, Fe_2_O_3_ NPs are prone to agglomeration or even clogging of vascular bundles, preventing their translocation to stems and leaves. As the concentration of Fe_2_O_3_ NPs increases, the chlorophyll content in plants decreases, and the MDA content significantly increases.

In summary, iron-based nanomaterials can generate hydroxyl radicals through Fenton or Fenton-like reactions, leading to changes in leaf antioxidant enzyme activity and malondialdehyde levels. Eventually, -OH promotes the degradation of chlorophyll, negatively impacting photosynthesis and thus inhibiting plant growth. No free iron ions were detected in the mixture of Fe_2_O_3_ NPs and chloroplasts, suggesting that the effect of free iron ions on -OH generation is negligible. Iron-based NMs show significant potential in enhancing chlorophyll content and antioxidant enzyme activity in Gramineae plants (Table 1). These materials, absorbed through the root system, not only promote chlorophyll synthesis and photosynthesis but also strengthen plant resistance to environmental stresses. However, the impact of iron-based NMs on the iron content in edible parts of Gramineae plants requires further study.

### 3.3. Impact of Titanium-Containing Nanomaterials on Gramineae Plants

Titanium dioxide nanoparticles (TiO_2_ NPs) are among the most produced NMs globally. Early research indicated that titanium application generally enhances plant enzyme activity and promotes the synthesis of crucial substances like chlorophyll and carotenoids, thus fostering plant growth. This effect is partly attributed to titanium and its compounds increasing iron activity in plant tissues, enhancing the absorption of elements like iron, magnesium, and phosphorus, thereby affecting plant metabolism. The transformation and translocation of TiO_2_ NPs within plant cells are usually achieved by adding TiO_2_ NPs to the water. However, high concentrations of titanium are toxic to plants [76]. High concentrations of TiO_2_ NPs can damage the photosynthetic pigments in plants. However, at low concentrations, TiO_2_ NPs, existing as metal oxides in the photosynthetic light reactions rather than as free metal ions, do not exhibit any toxic effects.

With advancing research on TiO_2_ NPs, it has been found that they can interfere in the early stages of plant growth. Through root exposure and foliar contact, TiO_2_ NPs affect the growth of seedlings. Morphological analysis revealed that titanium complexes with the parenchyma and vascular columns of common wheat accumulated in cells, endosperm, and nuclei. Once absorbed by seedlings, TiO_2_ NPs may cause metabolic anomalies, genotoxicity, and chloroplast structure damage [77]. The increase in TiO_2_ NP concentration leads to their extensive attachment to the plant root surface, blocking cell wall pores and causing osmotic stress due to reduced water absorption. This ultimately affects stomatal closure, reduces net carbon dioxide fixation, and inhibits plant transpiration. However, at suitable concentrations, the beneficial effects of titanium are more pronounced, especially in plants with nutrient deficiencies. For example, magnesium-deficient plants absorb titanium through roots, replacing phosphorus deposits in roots, thereby increasing the transport of magnesium to the plant apex. Foliar application of titanium improves seed germination [78]. This effect is attributed to the enhanced absorption of nitrates caused by TiO_2_ NP treatment, thereby accelerating chlorophyll synthesis and increasing the efficiency of solar energy capture, which in turn promotes photosynthesis. Studies on the physiological processes of rice seedlings show that TiO_2_ NP treatment significantly increases chlorophyll content and antioxidant enzyme activity [79]. Titanium application enhances plant absorption of elements like iron and magnesium, significantly increasing chlorophyll content, enzyme activities (like that of nitrate reductase), carotenoid content, photosynthesis rate, and symbiotic nitrogen fixation rate, accelerating rapid crop growth [80]. The promoting effect is often associated with increased activity of ribulose-1,5-bisphosphate carboxylase/oxygenase (RuBisCO) within the plant. TiO_2_ NPs induce changes in the secondary structure of enzymes, enhancing the activity of Mg^2+^-ATPase and chloroplast coupling factor (CF1)-ATPase on the one hand, improving light absorption and conversion efficiency, promoting carbon dioxide assimilation for plant growth [81]; on the other hand, they accelerate nitrogen metabolism within the plant, enhance the activity of enzymes such as nitrate reductase, and speed up the metabolic process of converting absorbed nitrates and inorganic nitrogen into organic nitrogen (such as proteins and chlorophyll), thereby promoting plant growth [82].

TiO_2_ NPs not only improve the absorption of plant nutrients but also alleviate the effects of cadmium, other nanoparticles, and drought stress. For example, plants exposed to TiO_2_ NPs exhibit higher levels of chlorophyll and carotenoids, showing a higher transpiration rate and less susceptibility to oxidative stress. However, some studies indicate the negative effects of TiO_2_ NPs on plant growth. Dias et al., using TiO_2_ NPs with rutile P25 long-term, found that P25-TiNP adversely affected normal wheat photosynthetic processes, reducing the content of substances like chlorophyll [83]. Xu et al.’s study showed that combined treatment of carbon dioxide with TiO_2_ NPs significantly increased chlorophyll and phosphorus content, plant height, and antioxidant activity in rice leaves as the CO_2_ concentration increased [84]. Additionally, foliar application of sodium nitroprusside protected wheat seedlings from drought-induced oxidative damage in environments exposed to TiO_2_ NPs [85]. Furthermore, the increase in proline and soluble sugar content in plant cells can effectively regulate the osmotic balance within plant cells under drought stress [86].

In conclusion, TiO_2_ NPs have various positive impacts on plants, including enhancing chlorophyll biosynthesis, improving photosynthetic efficiency, and stimulating nitrogen metabolism. TiO_2_ NPs also influence levels of plant growth hormones, protect plants from Cd absorption in polluted soils, and enhance nutrient absorption. However, under certain conditions, TiO_2_ NPs may produce harmful ROS, causing oxidative damage. Therefore, the application of TiO_2_ NPs for plant growth and development needs careful consideration.

### 3.4. The Impact of Nanozinc on Gramineae Plants

Zinc, a critical micronutrient for plant growth, is not only a vital component of enzymes like superoxide dismutase but also plays a key role in the repair process of photosystem II [87]. Zinc oxide nanoparticles (ZnO NPs) are extensively studied in agriculture, with both positive and negative effects on plants.

Studies by Boonyanitipong and Li, respectively, found that ZnO NPs negatively affect the growth of rice roots, potentially halting growth altogether [88,89]. Chen et al. further investigated the absorption and translocation of ZnO NPs in rice to reveal their toxicity mechanisms. Research has shown that under hydroponic conditions, the degradation rate of ZnO NPs is accelerated at the root, where they are absorbed in both ionic and particulate forms. It was found that the degradation rate of ZnO NPs in roots accelerates, as they are absorbed in both ionic and particulate forms. ZnO NPs inhibit the growth of rice seedlings by reducing biomass and chlorophyll content and decreasing the activity of antioxidant enzymes in rice, indicating potential oxidative damage caused by ZnO NPs [90]. Low concentrations of ZnO NPs enhance the synthesis of plant photosynthetic pigments and proteins, reduce the content of MDA, and alleviate membrane lipid peroxidation, whereas high concentrations of ZnO NPs disrupt chloroplast structure, decrease the number of grana and thylakoids, and reduce chlorophyll content. On the other hand, Meng’s study on the interaction between rice and metal oxide NPs (such as CuO NPs and ZnO NPs) revealed that under conditions of high exposure to MNPs, gene expression related to auxin biosynthesis in rice increased. These studies suggest that rice might increase its tolerance to MNPs by regulating auxin biosynthesis [91].

In practical agricultural applications, ZnO NPs are used as materials to improve crop growth conditions. Proper zinc addition can increase plant stress resistance. Mazhar et al. showed that treatment of rice seeds under drought conditions with ZnO NPs significantly increased chlorophyll contents and superoxide dismutase, catalase, and peroxidase activities, proving the effectiveness of ZnO NPs in improving crop growth under drought conditions [92]. Daniele et al. found that hybrid materials composed of ZnO-containing lignin nanoparticles (ZnO-L NPs) significantly increased the chlorophyll content and antioxidant properties of treated maize seeds [93]. Adil et al., applying ZnO NPs to wheat under salt stress, found that external application of ZnO NPs significantly improved the wheat’s chlorophyll content, plant height, and fresh root weight, achieving an increase in yield. ZnO NPs also enhance plant tolerance to salt and drought stress by improving plant nitrogen transport and total nitrogen content, regulating stress-related proteins, antioxidant enzyme activities, and stabilizing photosynthetic pigments [94]. Compared to traditional fertilizers, the external application of ZnO NPs had a more significant impact on the physical parameters and chlorophyll content of wheat [95].

However, it is important to note that zinc deficiency leads to slowed plant growth, reduced ability to cope with stress conditions, and decreased chlorophyll synthesis [87]. High concentrations of zinc can be toxic, similar to heavy metals, promoting ROS production and potentially displacing other metals from active sites in proteins, leading to iron and magnesium deficiency [96]. High concentrations of ZnO NPs can rapidly penetrate and accumulate in the vacuoles of plant root rhizomes, undergoing valence transformation (to Zn^2+^) and directly jeopardizing plant growth. ZnO NPs can positively affect the absorption and transfer of other nutrients, such as nitrogen, phosphorus, and boron, reducing the uptake of heavy metals. ZnO NPs have the advantage of slow dissolution compared to soluble salts, avoiding leaf burn, and converting into soluble salts within the plant, supporting gradual zinc absorption by the plant. In cases where rapid zinc absorption is required, the application of soluble zinc salts and chelates may be more suitable, while ZnO NPs can be used as slow-release zinc fertilizers. Foliar application of ZnO NPs can also reduce the uptake of heavy metals like cadmium, but there is a risk of reduced absorption of other nutrients like iron and magnesium.

### 3.5. The Impact of Nanocopper on Gramineae Plants

The chlorophyll content is a key determinant of plant photosynthetic capacity. Adequate copper (Cu) is beneficial for the formation and stability of chlorophyll, as Cu can form coordination compounds with chloroplast pigments [97]. However, high concentrations of Cu can be detrimental to plant chlorophyll content. This reduction in chlorophyll may be due to Cu entering the plant, causing an imbalance in chloroplast enzyme activity and accelerating chlorophyll degradation. Due to the difficulty in achieving uniform foliar application of NMs, treatment of Gramineae plants is usually conducted through hydroponic or soil application methods. CuO NPs can induce gradual deposition of lignin in roots, inhibiting root growth and obstructing the synthesis of abscisic acid and indole-3-acetic acid, leading to reduced absorption of trace elements (B, Mo, Mn, Mg, and Zn) by plants. Localized excessive accumulations of Cu may bind to the protein SH groups in chloroplasts or substitute for Fe^2+^ and Zn^2+^, causing changes and deactivation in the ionic composition of the chlorophyll–protein complexes. One of the reasons for the decline in photosynthetic ability is Cu^2+^ toxicity, which inhibits the transfer of photosynthetic electrons and the functionality of photosystem II [98]. CuO NPs negatively affect photosynthetic activity by deactivating the PSII reaction centers and reduce electron transfer, thylakoid number, photosynthetic rate, and photosynthetic pigments. With increasing Cu treatment concentration, the contents of chlorophyll a, chlorophyll b, and total chlorophyll in seedling leaves show a downward trend. Thus, Cu, as a key structural and catalytic component in various enzymes for electron transfer and redox reactions, is crucial for promoting plant growth. Cu mainly affects mitosis in plants, thus affecting the root system. Deposition of CuO NPs within leaf and root cells and on the surface of root tips leads to disordered arrangements of root tip cells, disconnection of cell wall linkages, loosening of the ties between cell wall microfibrils, disruption of cell adhesion, reduced levels of cell wall hemicellulose and esterified pectins, and swelling in the root hair zone, severely damaging the root system structure of the plant. These effects are likely caused by soluble Cu^2+^ ions. Most of the copper absorbed by plants from the soil accumulates in the roots, with relatively less transport to the above-ground parts [99]. However, the main reason for CuO NPs inhibiting plant growth is their induction of excessive ROS production within cells, which damages plant tissue cells, especially the root system, and significantly upregulates the expression of antioxidant enzyme-related genes in the oxidative defense system. This leads to protein dysfunction, adversely affecting Gramineae plants.

In practical applications, the effects of Cu NPs vary depending on their concentration and application method. For instance, Tamez’s soil application treatment of sugarcane with CuO NPs showed a significant increase in antioxidant enzyme activity and a slight increase in chlorophyll content [100]. Tiwari et al.’s treatment of rice seedlings with laser-ablated CuO NPs showed that low concentrations of CuO NPs promoted chlorophyll and antioxidant capacity in rice. In contrast, high concentrations showed obvious toxicity [2]. Choudhary et al. used chitosan copper nanoparticles to enhance maize’s defense response against Curvularia leaf spot (CLS) and promote plant growth [101]. Li et al. stimulated antioxidant enzymes in rice with MoS_2_-Cu NPs to achieve an antibacterial effect [102]. Notably, both Ag NPs and Cu NPs are heavy metal nanoparticles. Jaskulski’s application of urea foliar containing Ag NPs during barley cultivation showed that this urea could promote plant growth and increase antioxidant enzyme activity and chlorophyll content [103]. Therefore, combining urea or other substances with Cu NPs may reduce the oxidative damage of Cu NPs to plant cells and potentially confer certain resistance to the plant.

**Figure 6:**
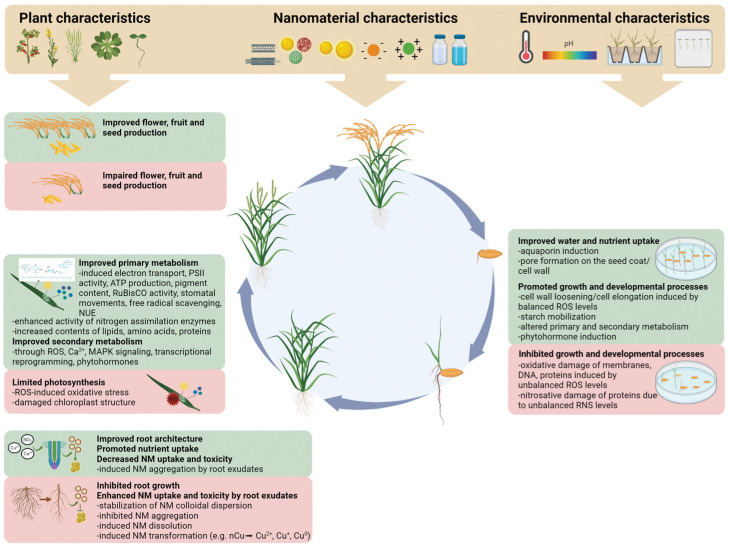
Positive and negative impacts of NMs on physiological processes throughout the plant lifecycle [104].

In conclusion, NMs have a wide-ranging impact on the physiological processes of plants throughout their lifecycle. These impacts can be positive or negative, depending on various factors, including plant characteristics (such as species and developmental stage), the chemical properties of NMs, particle size, concentration, and environmental conditions (Figure 6).

NMs can affect plant growth and development in multiple ways. For example, they can improve root structures and nutrient absorption, thereby enhancing overall plant health and growth rates. Additionally, controlling the use of NMs can reduce their toxic effects on plants, for instance, lowering the concentration of NMs or altering their chemical composition to mitigate negative impacts. However, the effects of NMs on plants are not always linear or predictable. Some types of NMs may have a promoting effect under certain concentrations or environmental conditions, while others may exhibit inhibitory or toxic effects. Therefore, comprehensive and detailed assessments of the potential impacts of NMs are required when applying them in agriculture and plant sciences. Moreover, with the ongoing development and application of nanotechnology, further research is needed into the long-term impacts of NMs on plant growth and environmental safety. Future research should focus on the environmental friendliness and sustainability of NMs to ensure that they enhance crop yield and quality without adversely affecting the ecosystem.

### 3.6. The Impact of Nanomaterials on the Environment

When assessing the application of NMs in agriculture and their ecological sustainability, it is crucial to delve into the potential impacts of these materials on non-target organisms, soil microbial communities, and the overall dynamics of ecosystems. NMs, due to their unique physical and chemical properties, such as their small size effects, surface effects, and quantum size effects, have been extensively researched and applied in various fields, including agriculture. However, these same properties have also raised concerns about environmental safety and ecological impacts. Firstly, NMs may have adverse effects on non-target organisms. These organisms are not the direct targets of nanomaterial applications but may be affected due to environmental exposure. For example, nanoparticles may accumulate through the food chain, affecting higher trophic levels [105]. Furthermore, the bioavailability and bioaccumulation of NMs may lead to unintended ecological risks. Secondly, soil microbial communities are a crucial component of agricultural ecosystems, responsible for nutrient cycling, organic matter decomposition, and soil structure maintenance. NMs could indirectly affect plant growth and soil health by altering the structure and function of soil microbial communities [106]. For instance, certain nanoparticles have been found to inhibit the growth of specific microbes, thereby disrupting the balance of microbial communities. Lastly, the impact of NMs on the overall dynamics of ecosystems is a complex issue that requires consideration of various ecological processes and interactions. NMs could alter how energy flows and material cycles in ecosystems, thereby affecting the stability and functionality of ecosystems [107].

**Figure 7:**
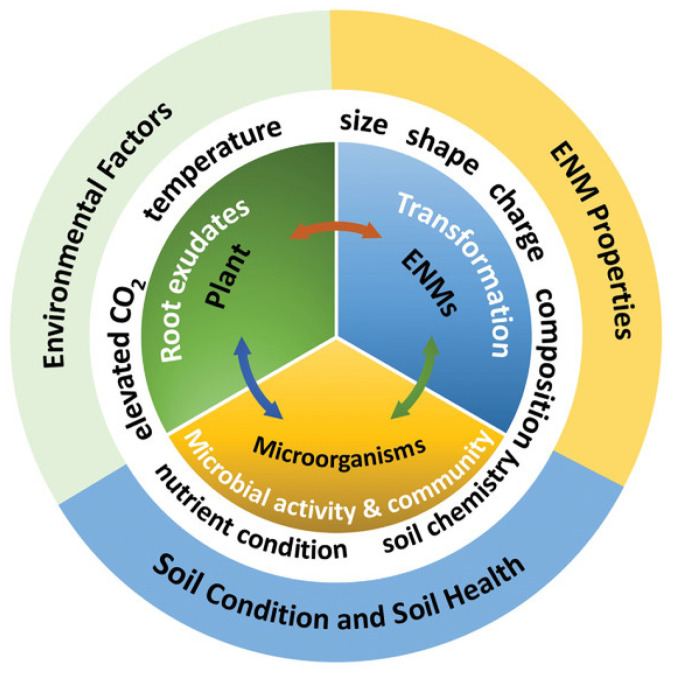
Transformation of ENMs in plants and microbes [109].

To unlock the full potential of nanotechnology in agriculture, it is essential to address the safety concerns associated with the use of NMs, with a key focus on understanding their transformation in the environment. The transformation of NMs is related to their physicochemical properties and dynamic changes in soil and biological environments. For example, MNPs (metal nanoparticles) in soil can rapidly dissolve, releasing metal ions that are directly accumulated by plants or forming complexes with other environmental components [108]. These factors are interconnected and influenced by environmental and climatic conditions. As shown in Figure 7, the interaction between plants, microbes, and NMs is complex [109]. Plants recruit specific microbial subgroups from the soil through root exudates, which settle and increase both inside and outside the root system, participating in carbon and nitrogen cycles, nitrogen fixation, and nutrient absorption, thus benefiting the plant [110]. NMs can alter microbial communities by affecting plant root exudates and extracellular substances, thereby impacting plant productivity. For instance, in one study, after the application of TiO_2_ NPs and ZnO NPs, the composition of bacteria involved in the nitrogen cycle significantly decreased. In contrast, bacteria involved in the decomposition of organic pollutants increased in the soil microbial community [111]. Moreover, titanium dioxide nanoparticles are known to affect the composition of mycorrhizal fungi related to wheat without negatively impacting plant growth [112]. However, the antimicrobial activity of NMs is also well documented; Johansen et al. found that the growth of fast-growing bacteria was inhibited by three to four times when the soil was treated with fullerenes 50 nm in size [113]. Among various CNTs, a reduction in GO had the most significant impact on microbial communities. NMs disrupt microbial cell membranes, protein denaturation, and DNA breakage in plant root microbiomes, interfering with microbial metabolic functions [114]. The interactions of NMs can affect the soil microbiome, the plant microbiome, and the overall health of the plant. Since the microbial communities of different soils and plants vary, generalizations cannot be made.

The application of nanotechnology in agriculture, especially nanofertilizers and nanopesticides, has shown potential to improve crop yield and resilience. However, it also raises concerns about potential impacts on the environment and ecosystems. Recent studies have begun to focus on the long-term effects of NMs on plant growth and soil health. For example, Upadhayay et al. explored the synergistic effects of NMs and plant probiotics in agriculture, emphasizing the potential benefits of their combined use for long-term agricultural sustainability. They noted that while the combined use of NMs and plant probiotics is in its early stages, it has shown better crop regulatory effects in enhancing crop productivity, alleviating environmental stress (such as drought, salinity, etc.), restoring soil fertility, and strengthening the economy. However, they also stressed the need for proper assessment before applying NMs to ensure that dosages do not produce any toxic effects on the environment and soil microbial communities [115]. Furthermore, Noor et al. studied the impact of organic and chemical fertilizers on soil health and the productivity of Taramira through long-term field experiments. They found that organic and integrated fertilizer options significantly enhanced plant growth characteristics, yield, and seed quality. This suggests that integrated fertilizer strategies, including the use of NMs, could be more beneficial due to their improving nutrient contents and energy values while effectively delaying plant senescence [116]. These studies indicate that the application of NMs in agriculture requires careful consideration of their long-term effects on crop productivity, soil health, and environmental safety. By conducting in-depth research and assessing the long-term effects of NMs, we can better understand how to utilize nanotechnology to enhance the sustainability and efficiency of agriculture without harming the environment and human health. The application of NMs in cereal crop production not only shows potential for improving crop yield and resilience but also raises concerns about their potential socio-economic impacts and equity issues.

Finally, the agricultural application of nanotechnology may lead to increased production costs and changes in production methods, impacting traditional agricultural knowledge and practices and thus affecting socio-economic welfare and ecosystem services [117]. Especially in developing countries, the inequality in technology access and benefit distribution could worsen, and small-scale and resource-poor farmers may struggle to afford the high costs of nanotechnology [118]. Therefore, ensuring that the application of nanotechnology in agriculture promotes social equity and economic welfare requires thoughtful planning and policy support to achieve equitable and sustainable technological development. This entails conducting independent impact assessments while promoting nanotechnology, ensuring community consent that is free, prior, and informed, and encouraging compliance with certification standards to increase the likelihood of project success and ensure fairness in agricultural investments.

## 4. Conclusions and Future Outlook

This study delves into the mechanisms of nanoparticle impacts on Gramineae plants, with a particular focus on three critical aspects: photosynthesis, chlorophyll content, and oxidative stress. We found that under certain concentrations, nanoparticles significantly promote the root development of Gramineae plants, thereby enhancing photosynthesis. This phenomenon is closely related to the increase in chlorophyll content, as chlorophyll is a core component of photosynthesis, and nanoparticles can effectively elevate its concentration under appropriate conditions. In terms of oxidative stress, the impact of NMs on Gramineae plants is distinct, as certain types of NMs can enhance the antioxidant enzyme activity of Gramineae plants, aiding their resistance to external environmental stresses. On the other hand, excessive NMs may lead to oxidative stress damage, thereby affecting plant photosynthesis and chlorophyll content. In particular, the metal ions produced by MNPs can damage plant cell structures, leading to oxidative stress. Therefore, the type and concentration of NMs have significant effects on the physiological and biochemical responses of Gramineae plants, necessitating further research for a deeper understanding.

This study provides an important theoretical foundation for the development of nanofertilizers that can precisely target Gramineae plants, enhance their antioxidant stress capacity, and improve photosynthesis efficiency. Future research should focus on designing and implementing more experiments to explore strategies that maximize the benefits of nanotechnology while minimizing its potential environmental impacts. In particular, the combined application of NMs with other substances to provide more favorable conditions for the growth and production of Gramineae plants warrants attention.

In summary, nanotechnology has shown tremendous potential in the growth and development of Gramineae plants. However, as its application continues to expand, we must cautiously address the potential environmental risks associated with nanotechnology. Ensuring that the increase in yield and quality of Gramineae crops goes hand in hand with maintaining the health and sustainability of the ecosystem will be a crucial topic in future nanotechnology research and application.

## Figures and Tables

**Table 1:** Influence of iron-based nanoparticles on the growth of Gramineae plants.

Material	Plant	Application Form	Effect	Proposed Mechanism of Action	Reference
nZVI, size of 33.8 ±3.59 nm	Rice	Soaking of seeds for 3 days in 20 mg/L suspension	Increased seedling growth	Chlorophyll content, NADPH dehydrogenase activity, and root metabolism significantly increased	[71]
nZVI or Fe_3_O_4_ NPs, size of 20 nm	Rice	Seedlings treated with 50 mg/L suspension for 14 days	Promoted seedling growth	Chlorophyll content and POD enzyme activity increased	[72]
Fe_3_O_4_ NPs, size of 50–100 nm	Rice	Spraying with 0–20 mg/L suspension for four months	Promoted growth of rice	Reduced chromium absorption and accumulation, chlorophyll content, and SOD enzyme activity	[73]
Fe_2_O_3_ NPs, size of 20–40 nm	Wheat	Seedlings treated hydroponically for 21 days	Root length, plant height, biomass, and chlorophyll content of wheat increased	NPs supported chlorophyll synthesis	[74]
Fe_3_O_4_ NPs, size of 6.85 ±1.70 nm	Wheat	Seeds treated with 2000 mg/L suspension for five days	Alleviation of heavy metal-induced oxidative stress in wheat seedlings	Absorption of cadmium, lead, copper, and zinc decreased, and antioxidant enzyme activities of SOD and POD increased	[75]
Fe_3_O_4_ NPs, size of 80–110 nm	Wheat	Treatment with 200–500 mg/L suspension for three weeks	Photosynthetic pigment content and SOD enzyme activity increased	Improved plant photosynthetic performance and iron and phosphorus utilization rates promoted plant growth	[57]

## Data Availability

The data that support the findings of this study are available from the corresponding author upon reasonable request.
